# Erianthridin suppresses non-small-cell lung cancer cell metastasis through inhibition of Akt/mTOR/p70^S6K^ signaling pathway

**DOI:** 10.1038/s41598-021-85675-8

**Published:** 2021-03-23

**Authors:** Sutthaorn Pothongsrisit, Kuntarat Arunrungvichian, Yoshihiro Hayakawa, Boonchoo Sritularak, Supachoke Mangmool, Varisa Pongrakhananon

**Affiliations:** 1 Department of Pharmacology and Physiology, Faculty of Pharmaceutical Sciences, Chulalongkorn University, 254 Phayathai, Wangmai, Pathumwan, Bangkok, Thailand; 2 Department of Pharmaceutical Chemistry, Faculty of Pharmacy, Mahidol University, Bangkok, Thailand; 3 Division of Pathogenic Biochemistry, Institute of Natural Medicine, University of Toyama, Toyama, Japan; 4 Department of Pharmacognosy and Pharmaceutical Botany, Faculty of Pharmaceutical Sciences, Chulalongkorn University, Bangkok, Thailand; 5 Department of Pharmacology, Faculty of Science, Mahidol University, Bangkok, Thailand; 6 Preclinical Toxicity and Efficacy Assessment of Medicines and Chemicals Research Cluster, Chulalongkorn University, Bangkok, Thailand

**Keywords:** Cancer, Drug discovery

## Abstract

Cancer metastasis is a major cause of the high mortality rate in lung cancer patients. The cytoskeletal rearrangement and degradation of extracellular matrix are required to facilitate cell migration and invasion and the suppression of these behaviors is an intriguing approach to minimize cancer metastasis. Even though Erianthridin (ETD), a phenolic compound isolated from the Thai orchid *Dendrobium formosum* exhibits various biological activities, the molecular mechanism of ETD for anti-cancer activity is unclear. In this study, we found that noncytotoxic concentrations of ETD (≤ 50 μM) were able to significantly inhibit cell migration and invasion via disruption of actin stress fibers and lamellipodia formation. The expression of matrix metalloproteinase-2 (MMP-2) and MMP-9 was markedly downregulated in a dose-dependent manner after ETD treatment. Mechanistic studies revealed that protein kinase B (Akt) and its downstream effectors mammalian target of rapamycin (mTOR) and p70 S6 kinase (p70^S6K^) were strongly attenuated. An in silico study further demonstrated that ETD binds to the protein kinase domain of Akt with both hydrogen bonding and van der Waals interactions. In addition, an in vivo tail vein injection metastasis study demonstrated a significant effect of ETD on the suppression of lung cancer cell metastasis. This study provides preclinical information regarding ETD, which exhibits promising antimetastatic activity against non-small-cell lung cancer through Akt/mTOR/p70^S6K^-induced actin reorganization and MMPs expression.

## Introduction

Lung cancer is one of the most frequently diagnosed cancers, and non-small-cell lung cancer (NSCLC) is responsible for 80–85% of all lung cancer cases. The prognosis of patients with advanced-stage cancer remains poor due to metastatic progression, and the 5-year survival rate is less than 6%^1^. Metastasis occurs through a multistep process in which cancer cells disseminate from the primary tumor by loss of cellular adhesion, which increases cell motility and invasiveness. Metastatic cancer cells enter the circulatory system, becoming circulating tumor cells, extravasate to distant organs and initiate secondary tumors^2^. Cell migration and invasion have been reported as early events that occur during the metastasis cascade, and blockade of these steps has been shown to suppress cancer metastasis^3^. Thus, attenuation of these aggressive behaviors provides an effective therapeutic strategy for advanced-stage cancer.

Cytoskeletal remodeling plays a crucial role in the morphological changes that occur during cell migration^4^. The polymerization of actin filaments at the front of the cell provides the formation of protrusive structures, including lamellipodia and filopodia, facilitating the attachment of the cells at the new site and providing a driving force for cell movement. Within the cell body, stress fibers (bundles of actin filaments) generate contractile forces to induce detachment of cells at the trailing edge^5^. The Rho family of small GTPases, including Cdc42, Rac1 and Rho A, has been reported to govern actin reorganization by hydrolyzing GDP to GTP^6^. Cdc42 and Rac1 promote the formation of filopodia (spike-like structures) and lamellipodia (flat sheet-like membrane protrusions consisting of actin polymers). Rho GTPases are responsible for modulating actin stress fibers, providing contractibility to cells during movement^6,7^. Recently, it has been demonstrated that PI3K/Akt/mTOR signaling is required for actin filament remodeling. Akt phosphorylation (p-Akt) or activation stimulates downstream effectors, including the mTOR and p70 S6 kinase (p70^S6K^) axis and cytoskeletal proteins^8^. Active p70^S6K^ enhances Rac1 and Cdc42 activation, causing polarized actin structures and directional cell migration. In addition, active p70^S6K^ binds to crosslinks filamentous actin (F-actin) to stabilize it by inhibiting cofilin-induced actin depolymerization^9^. Furthermore, the PI3K/Akt/mTOR signaling pathway is associated with the upregulation of matrix metalloproteinases (MMPs), including MMP-2 and MMP-9, which are enzymes required for ECM degradation^10,11^. Highly expressed MMP-2 and MMP-9 have been found in lung cancer tissue and are closely related to poor prognosis in patients with lung cancer. Targeting the Akt/mTOR/p70^S6K^ signaling pathway and actin reorganization are therefore promising approaches for attenuating cancer metastasis.

At present, therapeutic interventions mostly focus on localized cancer rather than migrating cells, and the repeat treatment cycle potentially mediates cancer aggressiveness phenotypes, which challenges anticancer drug discovery and development. Accumulating studies have demonstrated that various phenolic components derived from Thai orchids (*Dendrobium* spp.), such as moscatilin, gigantol and cypripedin, display anticancer properties, including apoptosis induction and inhibition of cell migration and cell invasion^12–14^. Erianthridin (ETD), a recently isolated phenolic compound from *Dendrobium formosum*, also exhibits several biological effects^15–17^. However, the effect of ETD on cancer metastasis remains unknown. In this study, we investigated the anti-metastatic effect of ETD in in vitro and in vivo models together with the molecular mechanism in non-small-cell lung cancer A549 and H460 cells.

## Results

### Cytotoxicity of ETD on non-small-cell lung A549 and H460 cancer cells

**Figure 1:**
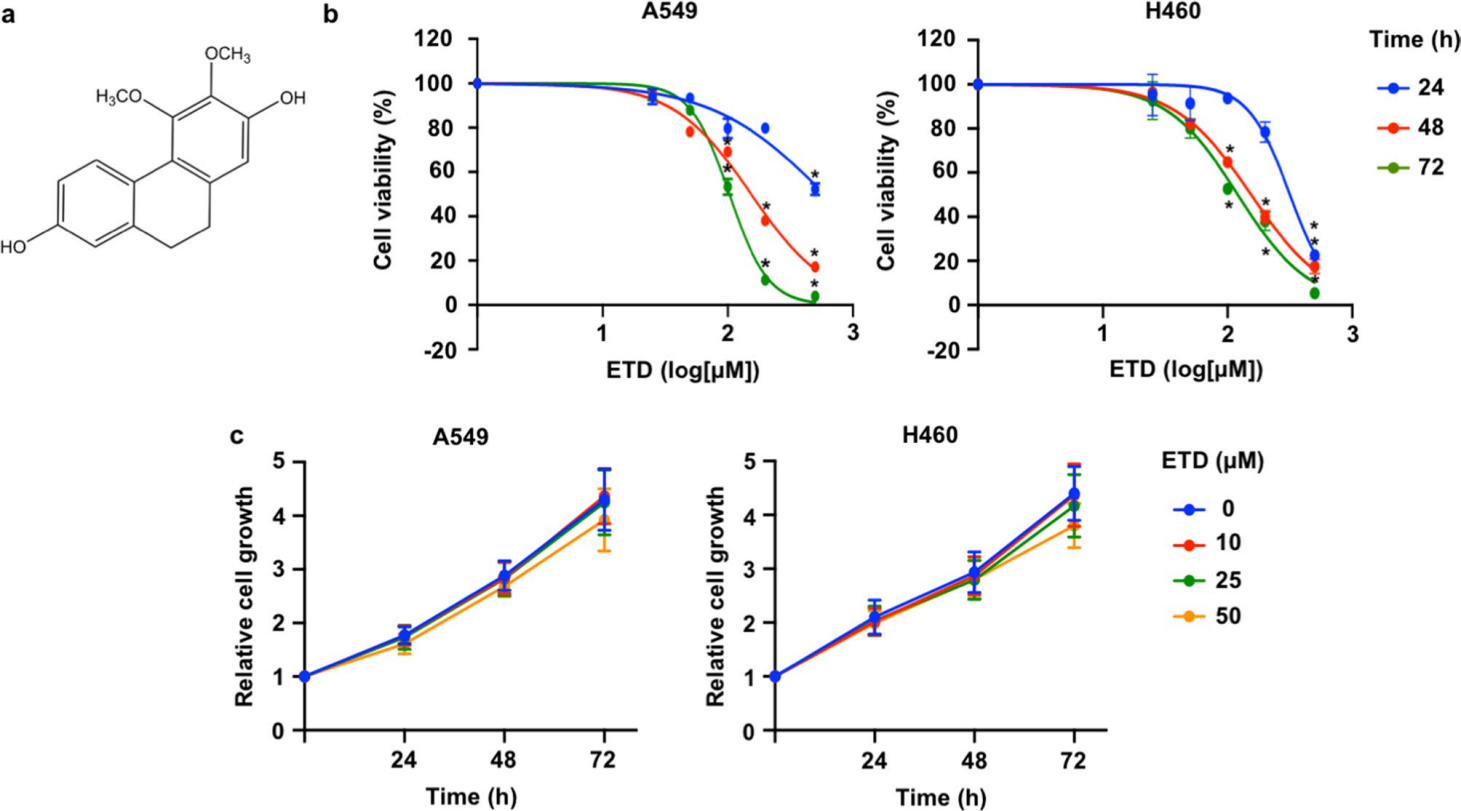
Cytotoxicity of erianthridin (ETD) in A549 and H460 cells. (**A**) The chemical structure of ETD (3,4-dimethoxy-9,10-dihydrophenanthrene-2,7-diol) is shown. (**B**) A549 and H460 cells were treated with the indicated concentrations of ETD for 24, 48 and 72 h. Cell viability was analyzed using the MTT assay and is represented as a percentage. (**C**) A549 and H460 cells were treated with nontoxic concentrations of ETD for 24, 48 and 72 h. Cell proliferation was evaluated by the MTT assay. The rate of cell growth was calculated as a value relative to time 0 h. The data are presented as the mean ± SEM (n = 3). *p < 0.05 vs untreated control cells.

We first assessed the cytotoxic effect of ETD on lung cancer cells. Human non-small-cell lung cancer A549 and H460 cells were treated with various concentrations of ETD (0–500 µM) for 24, 48 and 72 h. As observed in Fig. 1B, treatment with less than 50 µM ETD did not show cytotoxic effects in either type of lung cancer cell, whereas the higher concentration of ETD (≥ 100 µM) significantly reduced viable cells in a dose-dependent manner. Next, the effect of ETD on cell proliferation was determined. As seen in Fig. 1C, a nontoxic dose of ETD had no antiproliferative effect. Therefore, concentrations of ETD lower than 50 µM were employed in subsequent experiments to eliminate interference from the cytotoxic and proliferative effects of the compound on cell migration and invasion.

### ETD inhibits cancer metastatic behaviors in non-small-cell lung cancer cells

**Figure 2:**
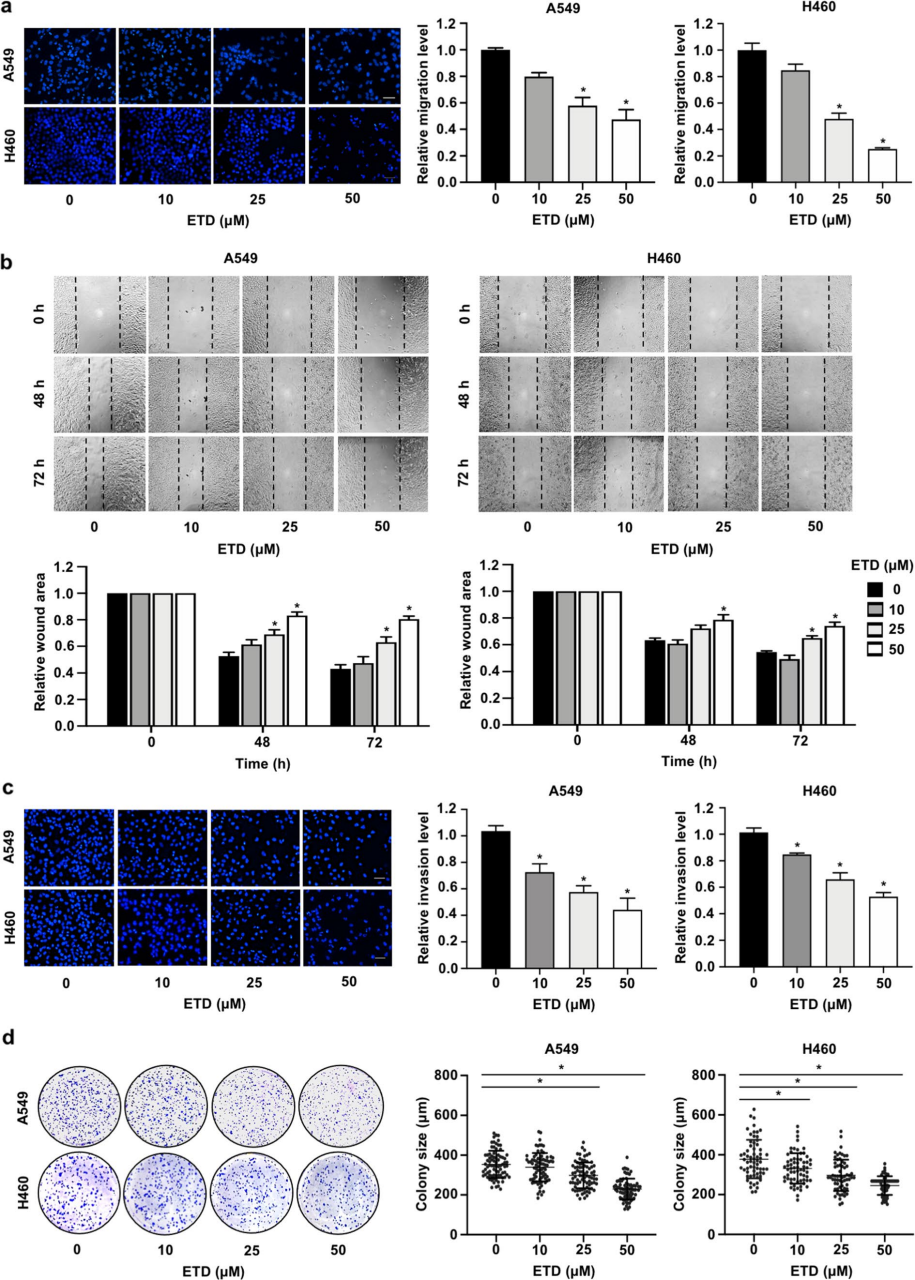
ETD inhibits metastatic behaviors of non-small-cell lung cancer cells. (**A**) A549 and H460 cells were seeded onto a transwell chamber and treated with nontoxic concentrations of ETD (0–50 µM). After 20 h, the migrated cells were stained with DAPI and imaged by fluorescence microscopy. The scale bar is 10 µm. (**B**) A monolayer of the cells was scratched with a pipette tip to generate a wound, and the cells were treated with 0–50 µM ETD. The wound area was photographed under a microscope at 0, 48 and 72 h. The wound area was quantified at each time point relative to the area at the initial time point. (**C**) A549 and H460 cells were seeded onto a transwell chamber coated with Martigel and treated with nontoxic concentrations of ETD (0–50 µM). The scale bar is 10 µm. (**D**) Anchorage-independent growth assays were conducted by seeding cells into 24-well plates coated with 0.5% agarose. Cells were incubated with ETD and allowed to grow for 10 d. The colony size was measured using ImageJ^54^. Each dot plot represents a single colony. All data are presented as the mean ± SEM (n = 3). *p < 0.05 vs untreated control group.

Since-cell migration and invasion are prerequisite steps in the metastasis process; to evaluate whether ETD can suppress metastatic cells, a transwell migration assay was performed. The results showed that ETD significantly reduced the number of migrated cells in a dose-dependent manner (Fig. 2A). Consistently, a wound healing assay was also conducted to evaluate the effect of ETD on collective cell migration in both cell types. As seen in Fig. 2B, migration was clearly decreased in the ETD-treated group, particularly with 50 µM ETD, compared with the control. To evaluate the anti-invasive effects of ETD, a transwell invasion assay was performed. The results demonstrated that 25 and 50 µM ETD extensively inhibited lung cancer cell invasion, with an inhibition rate of approximately 40–80% (Fig. 2C). Since cancer cells acquire survival mechanisms to overcome cell detachment-induced apoptosis during systemic circulation^18^, colony formation assays in soft agar were performed to explore the effect of ETD on anchorage-independent growth. The results showed that the colonies formed on soft agar were markedly smaller for cells treated with ETD than control cells, indicating that this compound was able to suppress anchorage-independent growth (Fig. 2D). These data suggest that ETD is an effective compound suppressing the metastatic behaviors of lung cancer cells.

### ETD suppresses cell migration and invasion via actin stress fiber reorganization and MMP inhibition

**Figure 3:**
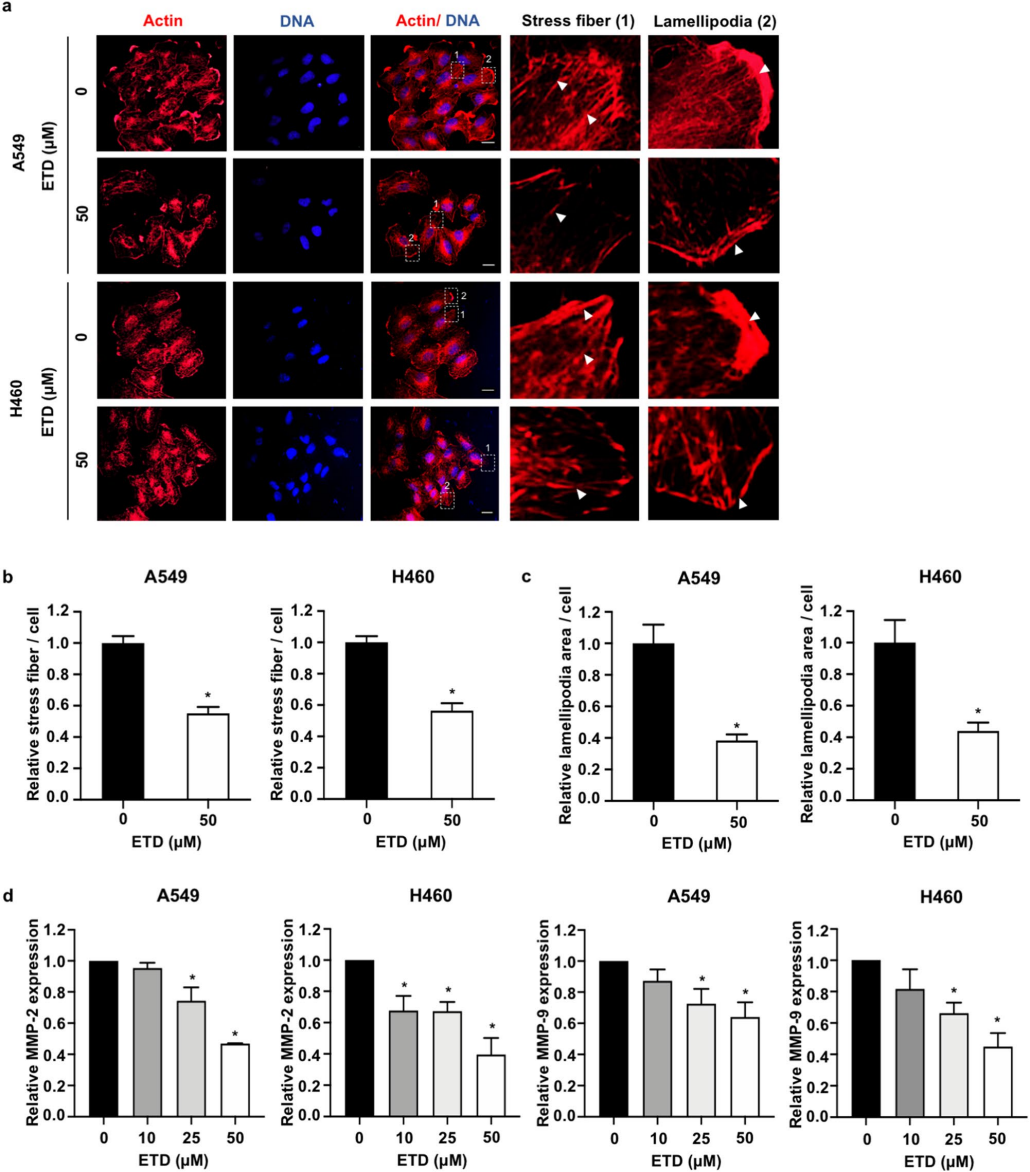
ETD suppresses cell migration and invasion via alteration of actin stress fiber organization and inhibition of MMP expression. (**A**) A549 and H460 cells were cultured on cover slips and treated with 50 µM ETD for 48 h. Cells were stained with phalloidin (actin, red) and DAPI (blue) and observed by confocal microscopy at ×20 magnification. The images in the box are enlarged in the right panel. Arrows indicate actin stress fibers and lamellipodia. The scale bar is 20 µm. (**B**) The number of stress fibers per cell and (**C**) the area of lamellipodia per cell were analyzed relative to those of the control group using ImageJ^54^. The data are presented as mean ± SEM from at least 50 cells. *p < 0.05 vs untreated control group. (**D**) Cells were treated with 0–50 µM ETD for 48 h, and MMP-2 and MMP-9 mRNA expressions were quantified by quantitative RT-PCR. The mRNA expression level of the treatment group was calculated relative to that in the control group. The data are presented as the mean ± SEM (n = 3). *p < 0.05 vs untreated control group.

Actin stress fibers, contractile actin bundles, are required for cell motility during metastasis^19^. Our results demonstrated that actin stress fibers were clearly present in nontreated cells, but they were markedly reduced after treatment with 50 µM ETD (Fig. 3A,B). We also investigated whether ETD-attenuated cell motility is involved in the alteration of lamellipodia formation. Immunofluorescence assay revealed that ETD effectively disrupted lamellipodial assembly (Fig. 3A). Quantitative analysis of lamellipodia demonstrated that the areas of lamellipodia formation in A549 and H460 cells were notably decreased in the presence of ETD (Fig. 3C). Since the Rac1 protein plays an important role in actin reorganization^20^, the active state of GTP-Rac1 was then examined. Western blot analysis showed that ETD strongly suppressed Rac1 activity (Supplementary Fig. S1), suggesting that ETD inhibited these actin dynamics in a Rac1-dependent manner. Furthermore, the mRNA levels of MMP-2 and MMP-9, extracellular matrix-degrading enzymes required for the cancer invasive process, were significantly downregulated by ETD in a dose-dependent manner (Fig. 3D). These data suggested that ETD suppresses lung cancer cell migration and invasion via alteration of actin organization and reduction of MMPs expression.

### ETD attenuates Akt/mTOR/p70^S6K^-mediated actin reorganization

**Figure 4:**
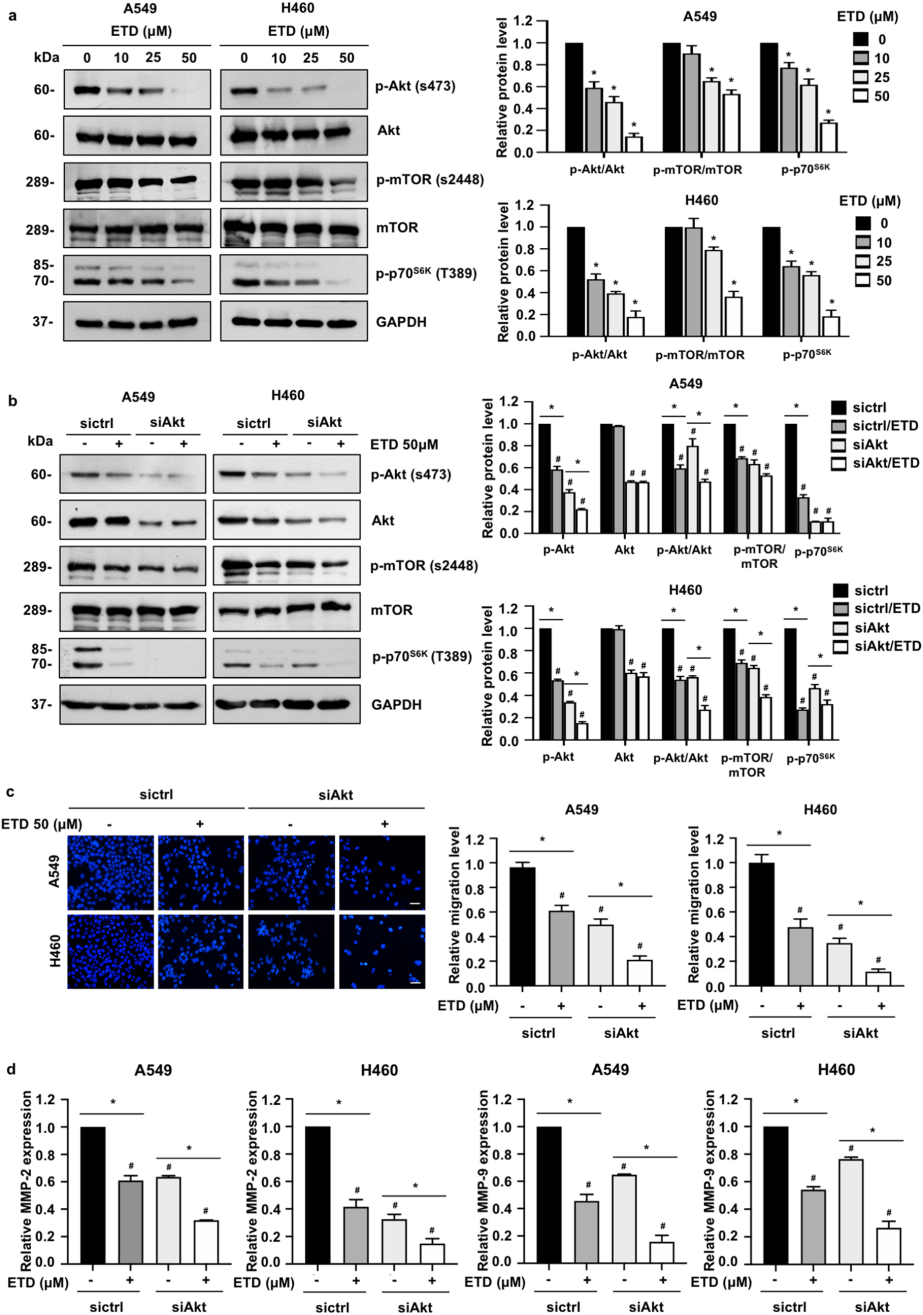
ETD inhibits cell migration and invasion via an Akt/mTOR/p70^S6K^-dependent mechanism. (**A**) A549 and H460 cells were treated with nontoxic concentrations of ETD for 24 h. The protein expression levels of p-Akt, Akt, p-mTOR, mTOR and p-p70^S6K^ were examined by Western blot analysis. The protein expression levels are displayed as the mean ± SEM (n = 3). *p < 0.05 vs untreated control group. (**B**) A549 and H460 cells were transfected with siRNA against Akt (siAkt) or si-mismatch control siRNA (siCtrl). After transfection for 18 h, cells were incubated with 50 µM ETD for 24 h and examined by Western blot analysis. The protein expression levels are shown as the mean ± SEM (n = 3). *p < 0.05 vs untreated cells, ^#^*p* < 0.05 vs untreated siCtrl cells. (**C**) A549 and H460 cells were transfected with siRNA against Akt (siAkt) or si-mismatch control siRNA (siCtrl). After transfection for 48 h, cells were subjected to transwell migration assay in the presence or absence of 50 µM ETD. The scale bar is 10 µm. The data are shown as the mean ± SEM (n = 3). *p < 0.05 vs untreated cells, ^#^*p* < 0.05 vs untreated siCtrl cells. (**D**) A549 and H460 cells were transfected with siRNA against Akt (siAkt) or si-mismatch control siRNA (siCtrl). After transfection for 18 h, cells were treated with 0–50 µM ETD for 48 h. MMP-2 and MMP-9 mRNA expressions were quantified by quantitative RT-PCR. The data are presented as the mean ± SEM (n = 3). *p < 0.05 vs untreated cells, ^#^*p* < 0.05 vs untreated siCtrl cells.

The Akt signaling pathway has been reported to control necessary cancer behaviors during metastasis, including cell motility and invasion^11^. To explore whether the Akt pathway is involved in ETD-mediated suppression of cell migration and invasion, Western blot analysis was performed. As seen in Fig. 4A, the level of Ser473-phosphorylated Akt (p-Akt), an active state of Akt, was gradually decreased in A549 and H460 cells treated with ETD, whereas total Akt was unchanged. Western blot analysis also revealed that ETD was able to downregulate the expression levels of p-mTOR (Ser2448) and p-p70^S6K^ (Thr389), members of a downstream signaling axis of Akt that regulates cell motility and invasion^21,22^, in A549 and H460 cells, suggesting that Akt/mTOR/p70^S6K^ signaling is a possible target by which ETD inhibits non-small-cell lung cancer cell migration and invasion.

To confirm whether the Akt pathway and its downstream effectors are required for the inhibitory effects of ETD on cell migration and invasion, we knocked down Akt in A549 and H460 cells by specific small interfering RNA (siAkt). The results demonstrated that the level of Akt, p-Akt and its downstream kinase (p-p70^S6K^) and p-mTOR were declined in response to siAkt transfection (Fig. 4B). The migrating cells and the mRNA levels of MMP-2 and MMP-9 were reduced accordingly (Fig. 4C,D). In addition, the reduction in p-Akt, p-p70^S6K^ and p-mTOR became more obvious after treatment with ETD in Akt knockdown cells. Together with, the suppression of cell migration and MMPs expression were potentiated by ETD treatment (Fig. 4C,D). These data supported our hypothesis that the Akt/mTOR/p70^S6K^ pathway participates in the ETD-induced attenuation of cell migration and invasion.

Epithelial-to-mesenchymal transition (EMT) has been reported to potentiate cancer cell movement^23^, and we tested whether ETD impedes the EMT process. Western blot analysis demonstrated that mesenchymal markers, including Snail, Slug and N-cadherin, were not significantly altered (Supplementary Fig. S2A). However, transforming growth factor β (TGF-β)-induced cell migration and anchorage-independent growth was extensively attenuated by ETD (Supplementary Fig. S2B–D). These data suggest that this inhibitory effect of ETD might function, in part, through suppression of Akt activity, since Akt participates in the noncanonical TGF-β pathway^24^.

### ETD directly binds to Akt via the protein kinase domain

**Figure 5:**
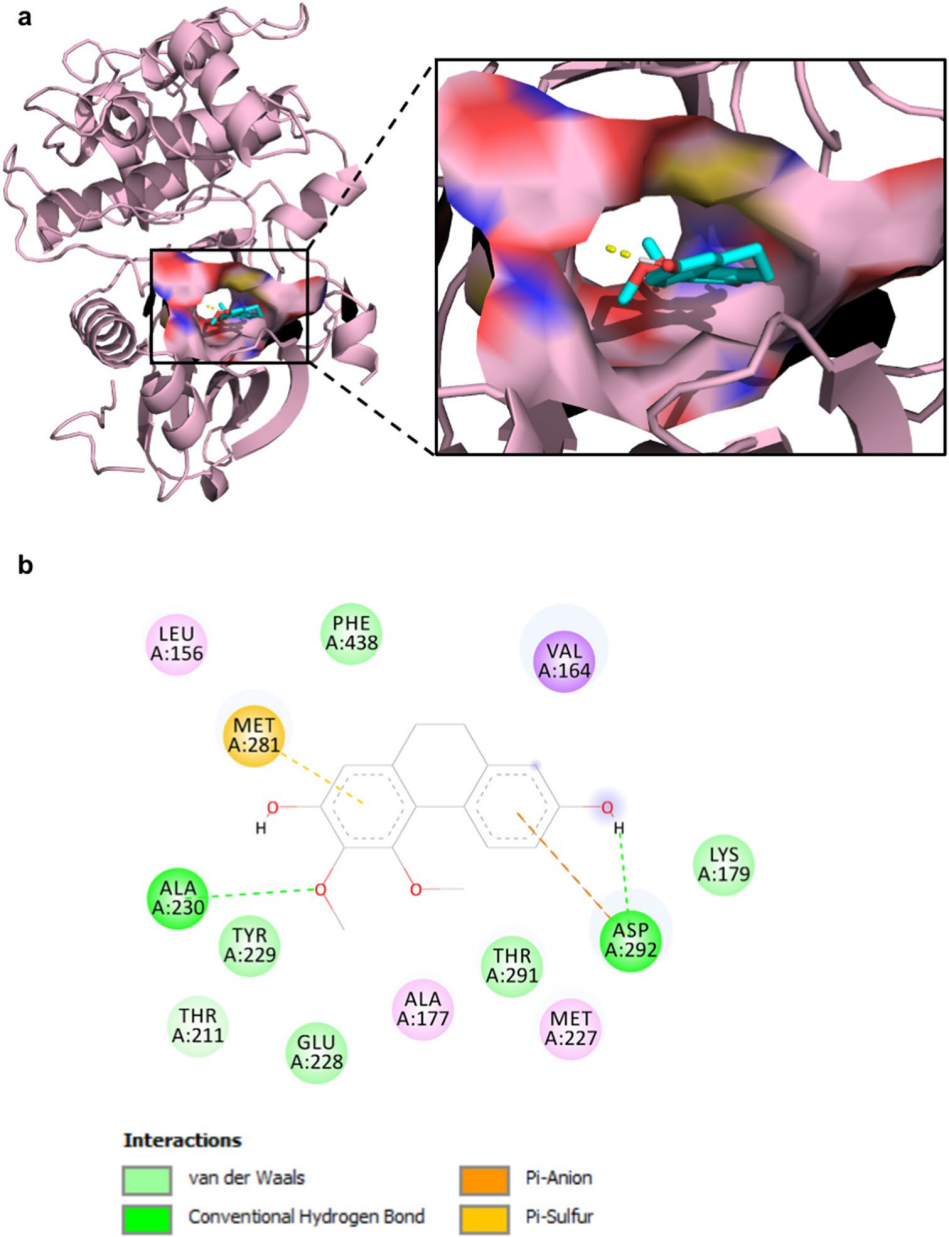
ETD directly binds to Akt. (**A**) Molecular docking analysis of ETD and Akt was performed by AutoDock4.2 and PyMOL program. (**B**) A schematic diagram, created by BIOVIA Discovery Studio Visualizer 2017, of the interacting residues between ETD and Akt is shown. Hydrogen bonds are displayed in green. The van der Waals interactions are shown in light green. The anion-π and sulfur-π interactions are shown in orange and yellow, respectively.

We further investigated whether Akt activity might be a result of direct interaction between ETD and Akt. A molecular docking study demonstrated that ETD binds to the ATP binding site in the protein kinase domain of Akt (Fig. 5A). The key interactions stabilizing the complex are hydrogen bonding and van der Waals interactions (Fig. 5B). The methoxy and phenol groups of ETD can form hydrogen bonds with the amide backbone of Ala230 (1.82 Å), which is a backbone amide in the kinase hinge, and the carboxylic acid side chain of Asp292 (1.93 Å), respectively. Other amino acids forming van der Waals interactions are Leu156, Val164, Ala177, Lys179, Thr211, Met227, Glu228, Tyr229, Met281, Thr291, and Phe438 (Supplementary Table S3). The methoxy group is oriented towards the gatekeeper Met227. Based on these interactions, the free binding energy between ETD and Akt is − 8.85 kcal/mol, and the ligand efficiency (LE) value of ETD is − 0.44 kcal/mol per heavy atom. These data indicate that ETD has a potential interaction with Akt and may interfere with Akt phosphorylation.

### ETD inhibits in vivo lung cancer metastasis

**Figure 6:**
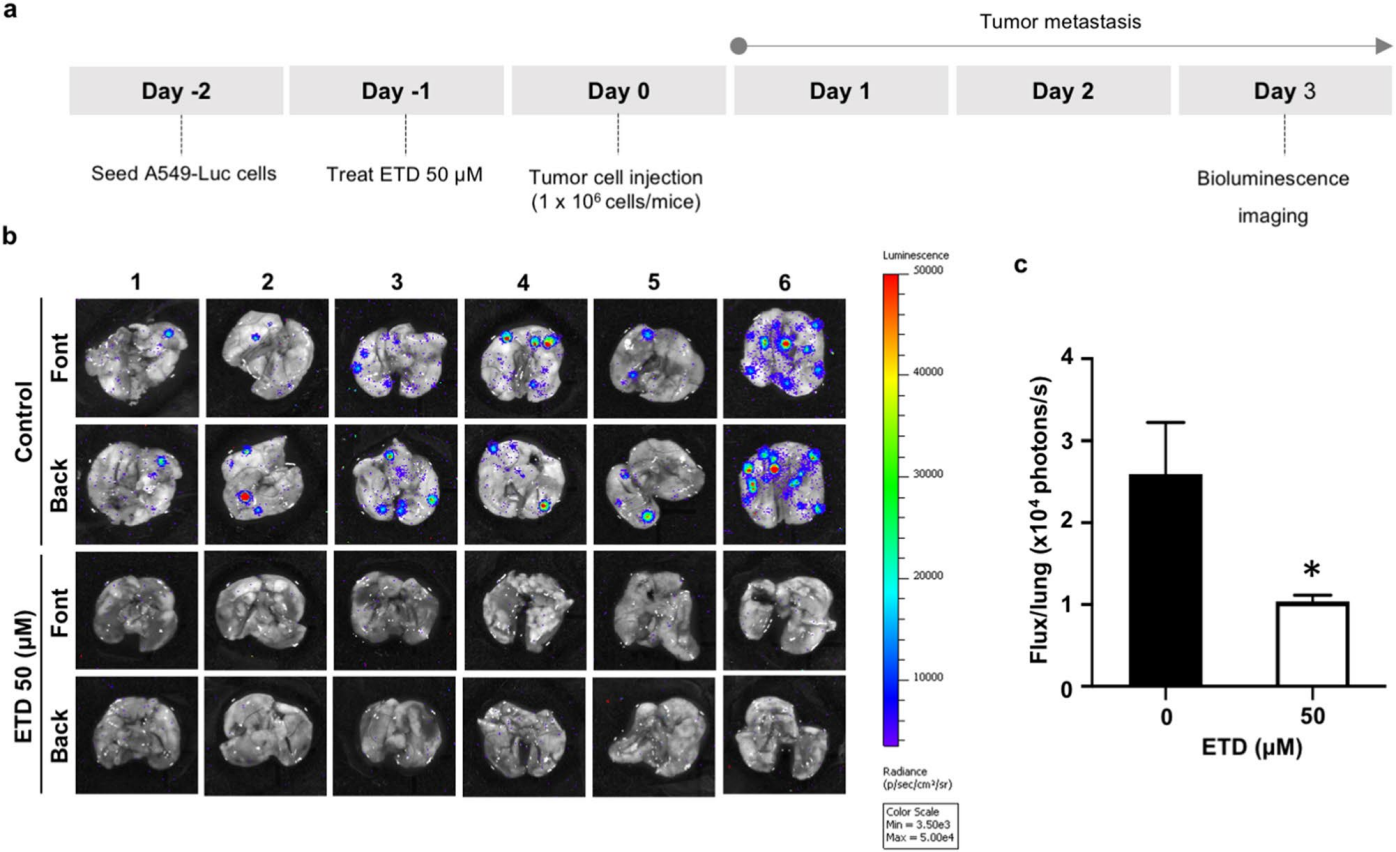
ETD attenuates an in vivo lung cancer metastasis. (**A**) Scheme for an in vivo lung cancer metastasis experiment. A549 cells expressing luciferase were treated with 50 µM of ETD for 24 h and injected into the tail vein of mice. (**B**) After 3 d of injection, the lung tissues were collected, and metastatic cancer cells were detected by IVIS imaging system. (**C**) Quantified values of bioluminescence intensity in ETD and control group are shown as a total flux (p/s). The data are presented as mean ± SEM (n = 6). *p < 0.05 vs untreated control group.

To confirm the role of ETD in lung tumor metastasis, A549-luciferase cells pretreated with 50 µM ETD were injected into the tail veins of mice. Three days after cell inoculation, the extent of lung metastasis was quantified by bioluminescence imaging (Fig. 6A). Pretreatment of cells with ETD demonstrated a significant reduction in metastatic foci (Fig. 6B). Quantitative analysis also revealed that compared with the control group, the group pretreated with ETD had remarkably suppressed lung cell metastasis (Fig. 6C), supporting the potent antimetastatic activity of ETD in lung cancer cells.

## Discussion

Lung cancer is one of the most serious malignancies worldwide due to its rapid metastasis^1^. Cell migration and invasion are recognized as critical steps in cancer metastasis, and the inhibition of these aggressive behaviors is of interest as a promising therapeutic approach. Previous studies have reported that phenolic compounds from *Dendrobium* spp*.* of Thai orchids exhibit antimetastatic activity via different molecular mechanisms^14,25,26^. In the present study, we first demonstrated the potent effect of ETD, a phenanthrene derivative isolated from Thai orchids, on suppressing lung cancer metastasis in both in vivo and in vitro studies. Furthermore, the underlying mechanism involved with the regulation of actin cytoskeleton rearrangement and MMP expression via the Akt/mTOR/p70^S6K^ signaling pathway.

The migration and invasion of cancer cells are hallmarks of malignancy, enabling cancer cell dissemination to distant organs^3^. It has been reported that reorganization of actin filaments is required for cancer cell migration and invasion^5,19^. Dynamic changes in the actin cytoskeleton promote the formation of discrete structures in cancer cells, including lamellipodia and stress fibers, which are essential for directional movement^19,27^. Several studies have demonstrated that disruption of actin structures is able to attenuate migration and invasion abilities in various cancer cell lines^28–30^, which is in agreement with our finding that the formation of stress fibers and lamellipodia was obviously disrupted in ETD-treated lung cancer cells and consequently resulted in decreased cell motility and invasion. Accumulating studies have revealed that Rac1, a member of the Rho family of small GTPases, participates in the organization of actin filaments and remodeling of the plasma membrane^20^. The GTP binding protein Rac1, in its active form, activates the Arp2/3 complex by binding with the SCAR/WAVE regulatory complex, which promotes the elongation of actin at the leading edge of motile cells^31^. Rac1 also functions as a direct regulator of actin stress fiber formation^32^. Overactivation of Rac1 has been found in various human cancers, including non-small-cell lung cancer^33^. The downregulation of Rac1 was shown to reduce the number of stress fibers^34^ and attenuate cancer cell migration and metastasis^35^. In agreement with our findings, the disruption of actin-based structures, including stress fibers and lamellipodia, is known to be related to a decrease in the active form of Rac1 in response to ETD treatment.

It is well known that PI3K/Akt signaling plays a dominant role in governing cancer cell migration and invasion. The activation of Akt participates in the reorganization of the actin cytoskeleton and mediates contraction of the cellular body through several downstream signaling pathways^36^. mTOR1, a downstream serine threonine kinase effector, was actively phosphorylated at Ser2448 by PI3K/Akt^37^. Loss of mTORC1 activity as a consequence of Akt inhibition contributed to a disruption of F-actin organization, including in lamellipodia and filopodia formation, at the leading edge of cancer cells^38^. In addition, p70^S6K^ is reported to be a downstream target of the PI3K/Akt/mTORC1 axis^39^. p70^S6K^ phosphorylated at Thr389 potently induces Rac1-mediated lamellipodia formation^9,36,40^. Inhibition of Akt/mTORC1/p70^S6K^ signaling resulted in an alteration of actin reorganization in favor of impeding cell motility^38^, suggesting an intriguing approach for attenuating cancer metastasis. Our findings also demonstrate that ETD significantly decreased Akt phosphorylation and activation of its downstream molecules mTOR and p70^S6K^, leading to the suppression of lung cancer cell migration. Furthermore, several studies have documented that activation of the PI3K/Akt/mTOR/p70^S6K^ signaling pathway triggers the expression of proteolytic enzymes facilitating cancer invasion, including MMP-2 and MMP-9^10,41^, and in particular, p70^S6K^ is an important transcription factor responsible for MMP-9 synthesis^41^. Based on this evidence and our finding, the reduction in MMP-2 and MMP-9 expressions induced by ETD in lung cancer cells is a consequence of inactivation of Akt and its downstream effectors.

By considering to the molecular structure of ETD, we further revealed how ETD has an inhibitory effect on Akt and whether there is an interaction among them. Akt consists of pleckstrin homology (PH), catalytic kinase, and regulatory domains, and its activity is regulated by phosphorylation and dephosphorylation processes in an Akt conformation-dependent manner. A recent study indicated that the quinone analog phenanthrene acts as a potent Akt inhibitor via direct interaction with Cys296 and Cys310 in a catalytic domain and induces Akt dephosphorylation^42^. Our study also demonstrated that ETD binds to the catalytic domain but at different sites. However, the interacting residues overlapped with those of the Akt inhibitors CID-20759629^43^ and A-674563^44^ (Supplementary Table S3), and the LE values were comparable to that in our study.

EMT is one of the crucial processes driving cancer metastasis. It involves genotypic and phenotypic changes of cells from an epithelial-like morphology to cells with loose cell–cell adhesion and a mesenchymal-like morphology^45^. Epithelial cells undergoing EMT decrease the expression of cell adhesion molecules, elevate the expression of mesenchymal markers and rearrange their cytoskeletons^45,46^. TGF-β, a multifunctional cytokine involved in many tumor cell functions, is a key modulator of the EMT mechanism^47^. The binding of TGF-β to its receptor initiates SMAD phosphorylation and activates downstream cascades in the canonical pathway^48^. TGF-β mediates EMT-associated transcription factors (TFs), including those of the Snail and the Slug families, and repressors of the E-cadherin promotor through SMAD signaling, which suppresses the expression of cell adhesion molecules^49^. In this study, we found that ETD was able to suppress TGF-β-induced metastatic phenotypes in A549 cells; however, ETD had no effect on Snail and Slug, a direct transcriptional repressor of E-cadherin, and N-cadherin expression (Supplementary Fig. S2). Since TGF-β-mediated EMT occurs through canonical and noncanonical pathways^24,50^, these data suggest that ETD diminishes TGF-β-induced metastatic phenotypes independent of canonical mechanisms. In addition, PI3K/Akt and the Rho GTPase family were reported to participate in a noncanonical pathway contributing to TGF-β-induced EMT^24,51^, suggesting that the inhibitory effect of ETD on TGF-β-enhanced cell migration is caused by ETD-induced suppression of Akt signaling and Rac1.

**Figure 7:**
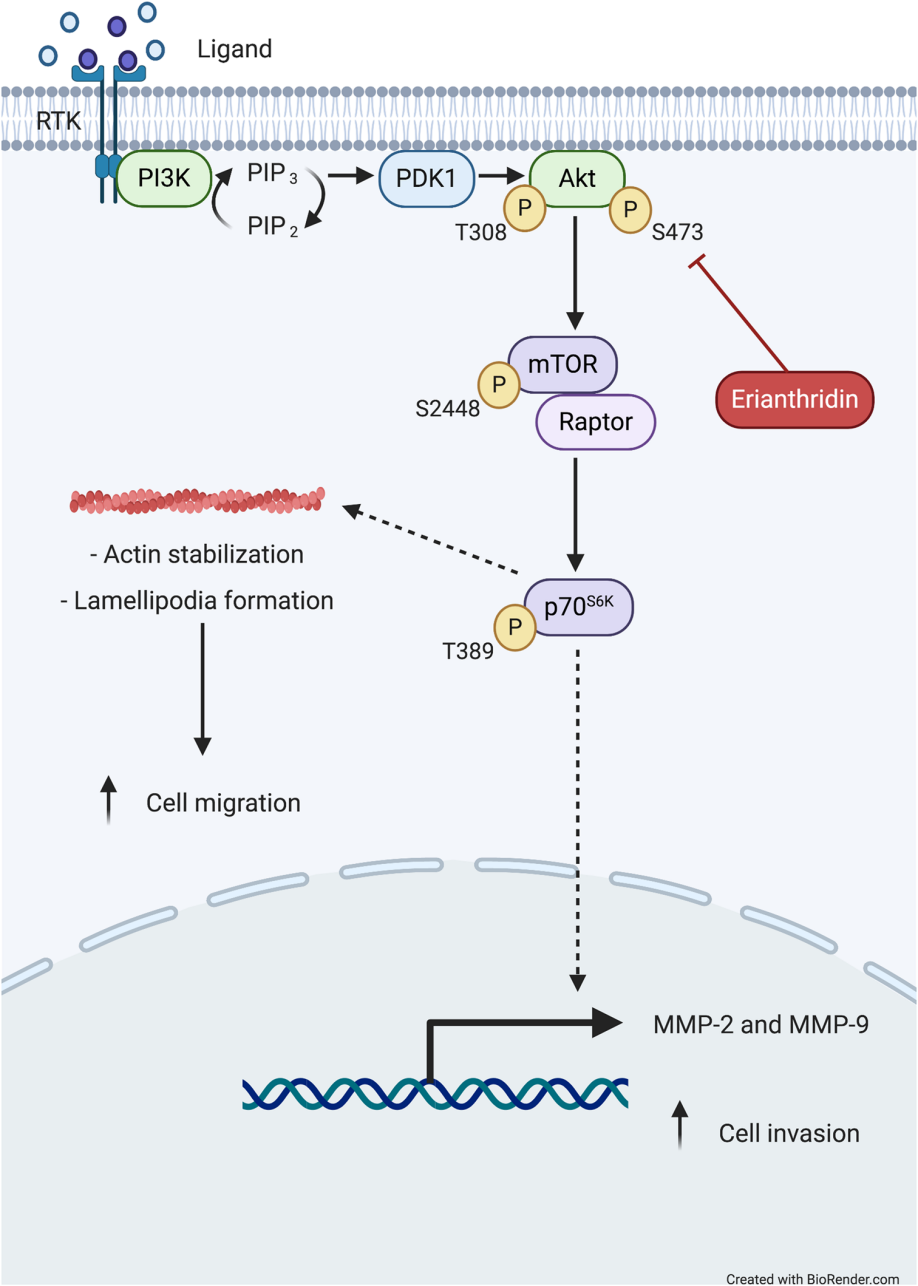
A Scheme diagram of this study. ETD suppresses lung cancer cell migration and invasion through inhibition of Akt/mTOR/p-p70^S6K^ signaling pathway. This diagram was created with “Biorender.com”.

In conclusion, this study demonstrated that ETD attenuates lung cancer cell metastasis in an in vivo and an in vitro studies. ETD exhibits an inhibitory effect on lung cancer cell migration and invasion via inhibition of Akt/mTOR signaling, and thereby modulates actin reorganization and downregulates MMP expressions (Fig. 7). This study suggests that the novel pharmacological activity of ETD warrants further research and development of this compound for ultimate use against non-small-cell lung cancer metastasis.

## Materials and methods

### Cell culture

Human non-small-cell lung A549 and H460 cells were obtained from American Type Culture Collection (ATCC) (Manassas, VA, USA). A549 and H460 cells were grown in DMEM and RPMI, respectively. Both of medium were supplemented with 10% fetal bovine serum (FBS), 100 U/mL penicillin–streptomycin and 2 mM L-glutamine. Cells were maintained in a 37 °C humidified incubator with 5% CO_2_. All media and supplements were purchased from GIBCO (Grand Island, NY, USA).

### Erianthridin preparation

ETD (Fig. 1A) was extracted from the whole plant of *Dendrobium formosum* Roxb. ex Lindl. as previously reported^52^. ETD was dissolved in dimethyl sulfoxide (DMSO) to make stock solution. The desired concentrations of ETD used were prepared by dilution with culture media. The final concentration of DMSO in all experiments was less than 0.1% which shows no observable toxic effect to cells.

### Cell viability assay

A549 and H460 cells (10^4^ cells/well) were seeded onto 96-well plates and incubated at 37 °C with 5% CO_2_ overnight. Then, cells were treated with various concentrations of ETD (0–500 µM) for 24, 48 and 72 h. Cell viability was examined by MTT assay as previously described^14^. The mean optical density in the indicated group was used to calculate the percentage of cell viability.

### Cell proliferation assay

A549 and H460 cells (2 × 10^3^ cells/well) were seeded onto 96-well plates and treated with non-toxic concentrations of ETD (0–50 µM) for 24, 48 and 72 h. At the end of incubation period, 10 μL of MTT solution (5 mg/mL) was added and incubated at 37 °C for 4 h. The medium was removed and replaced with 100 μL of DMSO to dissolve formazan crystals after incubation. The intensity of solution was measured at 570 nm by microplate reader. The absorbance in indicated group was calculated and represented as relative cell growth compared to control group.

### Wound healing assay

A549 and H460 cells (2 × 10^4^ cells/well) were seeded onto 96-well and incubated overnight. Cell migration was examined by Wound scratching assay as described^53^. The wound spaces were photographed under a phase contrast microscopy. The space area was quantified using ImageJ software (NIH)^54^ and represented as relative cell migration to the control group.

### Transwell migration and invasion assay

Transwell migration and invasion were determined using transwell chambers with and without Matrigel-coating. A549 and H460 cells (5 × 10^4^ cells/well) were seeded onto the upper chamber of 24-well transwell plates containing serum free media, and 600 μL media containing 10% FBS, a chemo-attractant, was added in the lower chamber. Cells were incubated for 18–20 h to allow cells movement into the underneath of membrane. Cells at the upper chamber were removed using cotton-swab, and migrating or invading cells at the lower surface of membrane were fixed with methanol and stained with DAPI. Cells from at least five random fields were imaged using fluorescence microscope (Nikon Inverted Microscope Eclipse Ti-U Ti-U/B, NY, USA) and presented as a relative value to the number of migrating or invading cells in the control group.

### Anchorage-independent growth assay

Anchorage-dependent growth was performed by maintaining the cells in soft agar as previously described^14^. Briefly, cells were treated with various non-toxic concentrations of ETD in 500 μL complete media by addition onto the upper layer of soft agar. At the end of incubation time, the colonies were stained with 0.01% crystal violet for 30 min at room temperature and washed with deionized water. All colonies per well were imaged under a phase contrast microscope. The size (μm) of the colonies was measured by ImageJ software (NIH)^54^.

### Immunofluorescence assay

A549 and H460 cells were plated at a density of 2 × 10^3^ cells onto the coverslip, and treated with non-toxic doses of ETD for 48 h. Immunostaining for actin was performed as described^14^. Cells were imaged using a fluorescence microscope (model IX81, Olympus, Japan). The number of actin stress fibers per cell and the extension of lamellipodia were analyzed by ImageJ software (NIH)^54^ in comparison to control group.

### Quantitative real-time PCR (qRT-PCR)

A549 and H460 cells were treated with non-toxic doses of ETD for 48 h. Total RNA was extracted using the Qiagen RNeasy kit (Qiagen, Valencia, CA, USA) following the manufacture’s instruction. The qualitative real-time PCR was performed for MMP-2 and MMP-9 expressions using One step TB Green PrimeScript PLUS RT-PCR Kit (Takara, Japan). The primers used were listed in Supplementary Table S2. The expression levels of the target genes were calculated using (2^−ΔΔCt^) method.

### Western blot analysis

A549 and H460 cells were treated with non-toxic doses of ETD and incubated for 24 h. At the end of incubation, cells were lysed in TMEM lysis buffer as described^53^. An equal protein content was dissolved by SDS–polyacrylamide gels and electrotransferred onto polyvinyl difluoride (PVDF) membranes. The membranes were blocked, incubated with specific primary and secondary antibodies as described in Supplementary Table S1. The blots were visualized by enhanced chemiluminescence system using Immobilon Western chemiluminescent HRP substrate (Millipore, MA, USA). GAPDH was used as a loading control. Quantification of the band intensity of protein expression was performed using ImageJ software (NIH)^54^.

### Small interference RNA Transfection assay

The transfection of small interfering RNA (siRNA) targeting Akt was performed as described using Lipofectamine RNAiMAX (Invitrogen, Carlsbad CA, USA)^14^. The siRNA sequences are following: siAkt, sense 5′-GGAGAUCAUGCAGCAUCGC-3′ and anti-sense: 5′-GCGAUGCUGCAUGAUCUCC-3′; si-mismatch control, sense 5′-GGGAAUCAUAA- AGCAUUUC-3′ and anti-sense 5′-CCGGGGCUGCAUAAACUUC-3′.

### In vivo tail vein metastasis assay

Five to six-week old CB17-Prkdc^scid^ mice were obtained from the CLEA Japan, Inc. (Tokyo, Japan) and maintained under specific pathogen-free conditions throughout the study. The mice were randomly separated into six mice in each group. A number of 10^6^ A549-luc cells, that were incubated with or without 50 μM ETD for 24 h, were injected into tail vein of mice. The mice were sacrificed after injection 3 d, and lung metastasis was evaluated by IVIS Lumina II System (Caliper Life Science, MA, USA).

### Molecular docking

The X-ray crystal structure of Akt was retrieved from Protein Data Bank (PDB) with PDB ID 3MVH. The structure of ETD was drawn by ChemDraw Ultra 17.0 (PerkinElmer, Waktham, MA, USA). The molecular docking of ETD and Akt was performed by AutoDock4.2^55^, which genetic algorithm (GA) parameters included 100 GA runs, a popular size of 150, a maximum of 10,000,000 evaluations, and a maximum of 27,000 generations as described in previous study^56^. The 3D ligand conformations, aligned within 2.0 Å root-mean-square deviation (RMSD), were grouped as the same conformation clusters. The ligand conformations which have the highest cluster were analyzed for free binding energies (ΔG) and ligand efficiency (LE). The binding interaction between ligands and target protein was analyzed by AutoDock4.2, PyMOL (Schrödinger, New York, NY, USA) and BIOVIA Discovery Studio Visualizer 2017 (Biovia, San Diego, CA, USA). The parameters and analysis were described in Supportive Information.

### Statistical analysis

Data are presented as mean ± S.E.M at least three-independent experiments, and all data were analyzed using Prism 8 (GraphPad Software, Inc., San Diego, CA, USA). The student’s *t*-test was used to analyze statistical differences between two groups. The One-way ANOVA with Tukey’s Multiple Comparison Test was applied for determination the statistical significance between control and treatment groups. *P-*values less than 0.05 were considered statistically significance.

### Ethic statement

All protocols were performed in accordance with relevant guideline. Animal experiments were approved by the Animal Experiment Ethics Committee of the University of Toyama (A2019INM-5), and were carried in accordance with the ARRIVE guidelines.

## Supplementary Information

Supplementary Information.
