# Supplementary material for: Erianthridin suppresses non-small-cell lung cancer cell metastasis through inhibition of Akt/mTOR/p70S6K signaling pathway
Source: Sci Rep. 2021 Mar 23;11:6618. doi: 10.1038/s41598-021-85675-8 (PMC7987990; doi:10.1038/s41598-021-85675-8)
Supplement: Supplementary file 1 — Supplementary Information. [file 41598_2021_85675_MOESM1_ESM.pdf]

Supplementary information

Erianthridin suppresses non-small-cell lung cancer cell metastasis through inhibition of Akt/mTOR/p70^{S6K} signaling pathway

Sutthaorn Pothongsrisit[1], Kuntarat Arunrungvichian[2], Yoshihiro Hayakawa[3], Boonchoo Sritularak[4], Supachoke Mangmool[5], Varisa Pongrakhananon[1,6,*]

[1]Department of Pharmacology and Physiology, Faculty of Pharmaceutical Sciences, Chulalongkorn University, Bangkok, Thailand

[2]Department of Pharmaceutical Chemistry, Faculty of Pharmacy, Mahidol University, Bangkok, Thailand

[3]Division of Pathogenic Biochemistry, Institute of Natural Medicine, University of Toyama, Toyama, Japan

[4]Department of Pharmacognosy and Pharmaceutical Botany, Faculty of Pharmaceutical Sciences, Chulalongkorn University, Bangkok, Thailand

[5]Department of Pharmacology, Faculty of Science, Mahidol University, Bangkok, Thailand

[6]Preclinical Toxicity and Efficacy Assessment of Medicines and Chemicals Research Cluster, Chulalongkorn University, Bangkok, Thailand

***Corresponding author**

Varisa Pongrakhananon, Department of Pharmacology and Physiology,

Faculty of Pharmaceutical Sciences, Chulalongkorn University, 254 Phayathai, Wangmai, Pathumwan, Bangkok, Thailand 10330

Tel: +662-218-8325; Fax: +662-218-8340

Email: Varisa.p@pharm.chula.ac.th

Supplementary methods

Rac1 activity assay

Active Rac1 (GTP-bound) was isolated using the Rac1 activation assay kit (Cell Biolabs, Inc., Japan) following the manufacturer's instructions. Briefly, GST-PAK-1 PBD fusion protein were added to the lysate and gentle agitated for 1 h at 4° C. The beads were washed with 1X assay lysis buffer and pelleted by centrifugation for 10 sec at 14,000 x g. The beads were resuspended in reducing SDS-PAGE sample buffer and subjected to immunoblotting using specific antibody to active GTP-Rac1.

Table S1 List of antibodies used in this study.

Antibodies	Dilution	Company
rabbit anti-phosphorylated Akt (S473)	1:1000	Cell signaling # 9271
rabbit anti-Akt	1:1000	Cell signaling # 9272
rabbit anti-phosphorylated mTOR	1:1000	Cell signaling # 5536
rabbit anti-mTOR	1:1000	Cell signaling # 2938
rabbit anti-phosphorylated p70 S6 kinase (Thr389)	1:1000	Cell signaling # 9234
rabbit anti-N-cadherin	1:1000	Cell signaling # 13116
rabbit anti-Slug	1:1000	Cell signaling # 9585
rabbit anti-Snail	1:1000	Cell signaling # 3879
mouse anti-Rac1	1:1000	Cell biolabs # 240106
anti-rabbit IgG HRP-linked	1:1000	Cell signaling # 7074
anti-mouse IgG HRP-linked	1:1000	Santa Cruz # sc-516102
Alexa Fluor 568 phalloidin	1:1000	Life technologies # A12380

Table S2 List of primers used in this study.

Name	Sequence
MMP-2	Forward: 5’-GAA GTA TGG GAA CGC CGA TGG-3’<br>Reverse: 5’-TTG TCG CGG TCG TAG TCC TCA-3’
MMP-9	Forward: 5’-CCT GGA GAC CTG AGA ACC AAT C-3’<br>Reverse: 5’-CCA CCC GAG TGT AAC CAT AGC-3’
GAPDH	Forward: 5’- ACA TCG CTC AGA CAC CAT G -3’<br>Reverse: 5’- TGT AGT TGA GGT CAA TGA AGG G -3’

Table S3 The interaction strength of ETD, CID-20759629 and A-674563 with Akt.

Compound	Binding Energy	Ligand efficiency	Interaction	
			H-bond	Van der Waals
ETD	-8.85	-0.44	Asp292<br>Ala230	Leu156<br>Val164<br>Ala177<br>Lys179<br>Thr211<br>Met227<br>Glu228<br>Tyr229<br>Met281<br>Thr291<br>Phe438
CID-20759629	-11.97	-0.50	Ala230	Leu156<br>Gly157<br>Val164<br>Ala177<br>Met227<br>Tyr229<br>Ala230<br>Met281<br>Thr291<br>Phe438<br>Phe442
A-674563	-14.32	-0.48	Glu288<br>Asp292	Leu156<br>Gly159<br>Gly162<br>Val164<br>Ala177<br>Lys179

				Leu181<br>Met227<br>Glu228<br>Met281<br>Thr291<br>Asp292<br>Phe438

Supplementary figures

Supplementary Fig S1

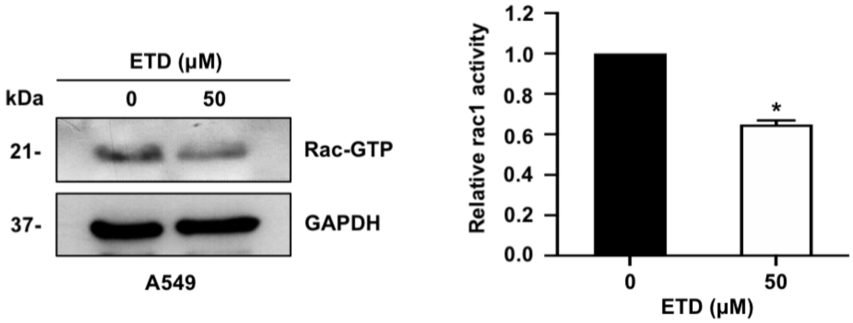

Figure S1 ETD inhibit Rac1 activity. A549 were treated with 50 μM of ETD for 48 h. Rac1 activity was determined by Rac1 activation assay kit. GADPH was reprobed as a loading control. The intensity was qualified and normalized with loading control by ImageJ[1]. The level of Rac1 activity were plotted as as mean ± SEM (n = 3). *p < 0.05 vs untreated control group.

Supplementary Fig S2

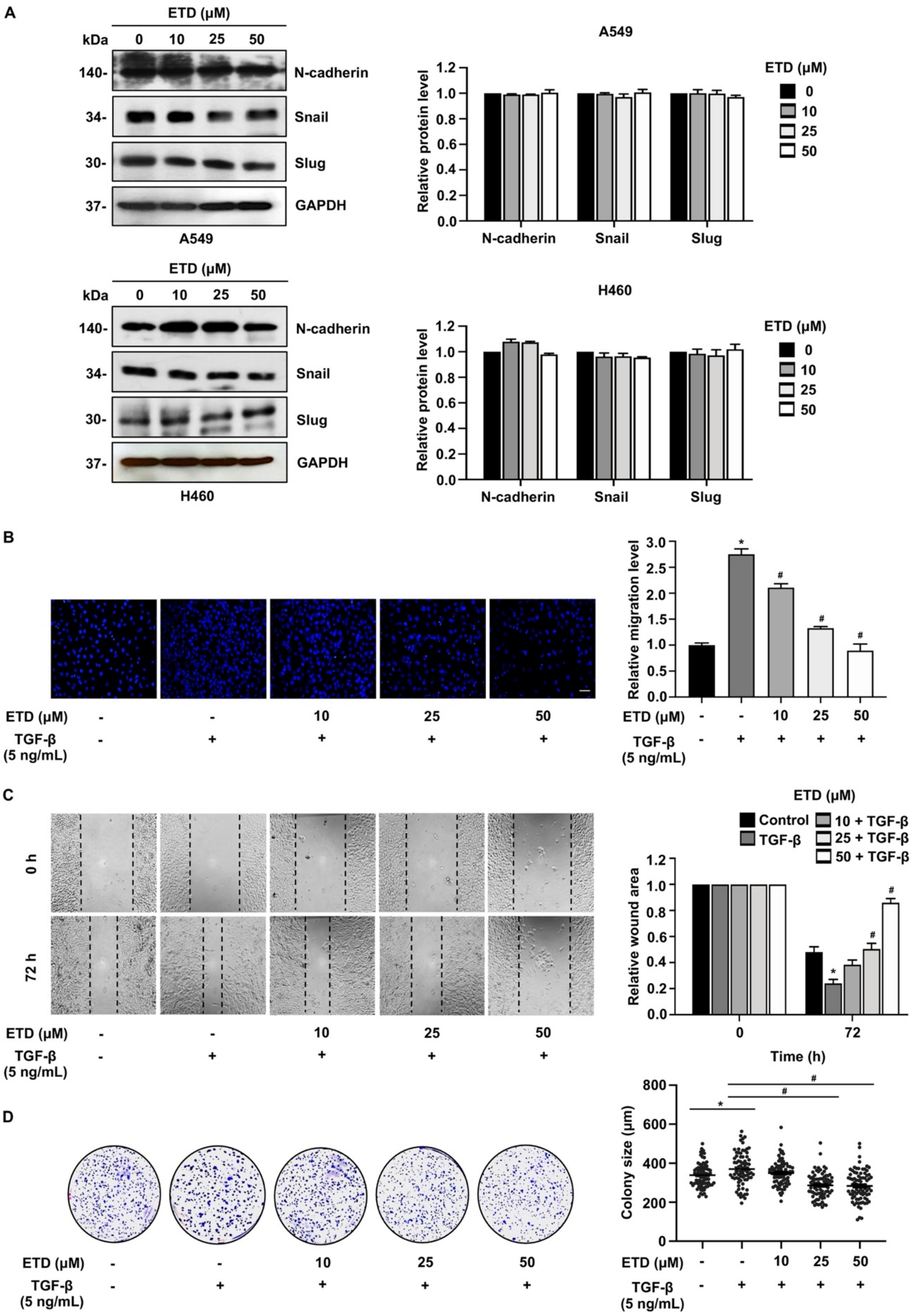

Figure S2 ETD attenuates TGF-β-induced epithelial-to-mesenchymal phenotypes. (**A**) A549 and H460 cells were treated with non-toxic concentrations of ETD (0-50 μM) for 24 h. The protein expression level of N-cadherin, snail and slug were examined by Western blot analysis. GADPH was reprobed as a loading control. The intensity was qualified and normalized with loading control by ImageJ. The protein expression levels were plotted as as mean ± SEM (n = 3). *$p < 0.05$ vs untreated control group. (**B**) A549 cells were seeded onto the transwell chamber and incubated with non-toxic concentration of ETD (0-50 μM) for 8 h followed by treatment with TGF-β (5 ng/ml) for 12 h. The migrated cells were stained with DAPI and imaged by fluorescence microscopy. The cells on the lower side of transwell were counted and calculated as the relative number of migrated cells of treatment group compared to control group. The data are presented as mean ± SEM (n = 3). *$p < 0.05$ vs untreated control group. $^{\#}p < 0.05$ vs TGF-β treated alone. Scale bar is 10 μm. (**C**) Monolayer of the cells was scratched with pipette tip to generate wound space and pretreated with 0-50 μM of ETD for 8 h followed by treatment with TGF-β (5 ng/ml). The wound area was photographed under microscope at 0, 72 h. The wound space was quantified as an area at each time point relative to an area at initial time point. The data are presented as mean ± SEM (n = 3). *$p < 0.05$ vs untreated control group. $^{\#}p < 0.05$ vs TGF-β treated alone. (**D**) Anchorage-independent growth assay were conducted by seeding cells onto 24-well coated with 0.5 % agarose. Cells were incubated with ETD for 8 h followed by treatment with TGF-β (5 ng/ml) and allowed for growing 10 d. The colonies were stained with crystal violet, and the colony size was measured using ImageJ[1]. Each dot plot represented a single colony. All data are presented as mean ± SEM (n = 3). *$p < 0.05$ vs untreated control group. $^{\#}p < 0.05$ vs TGF-β treated alone.

Supplementary Fig S3

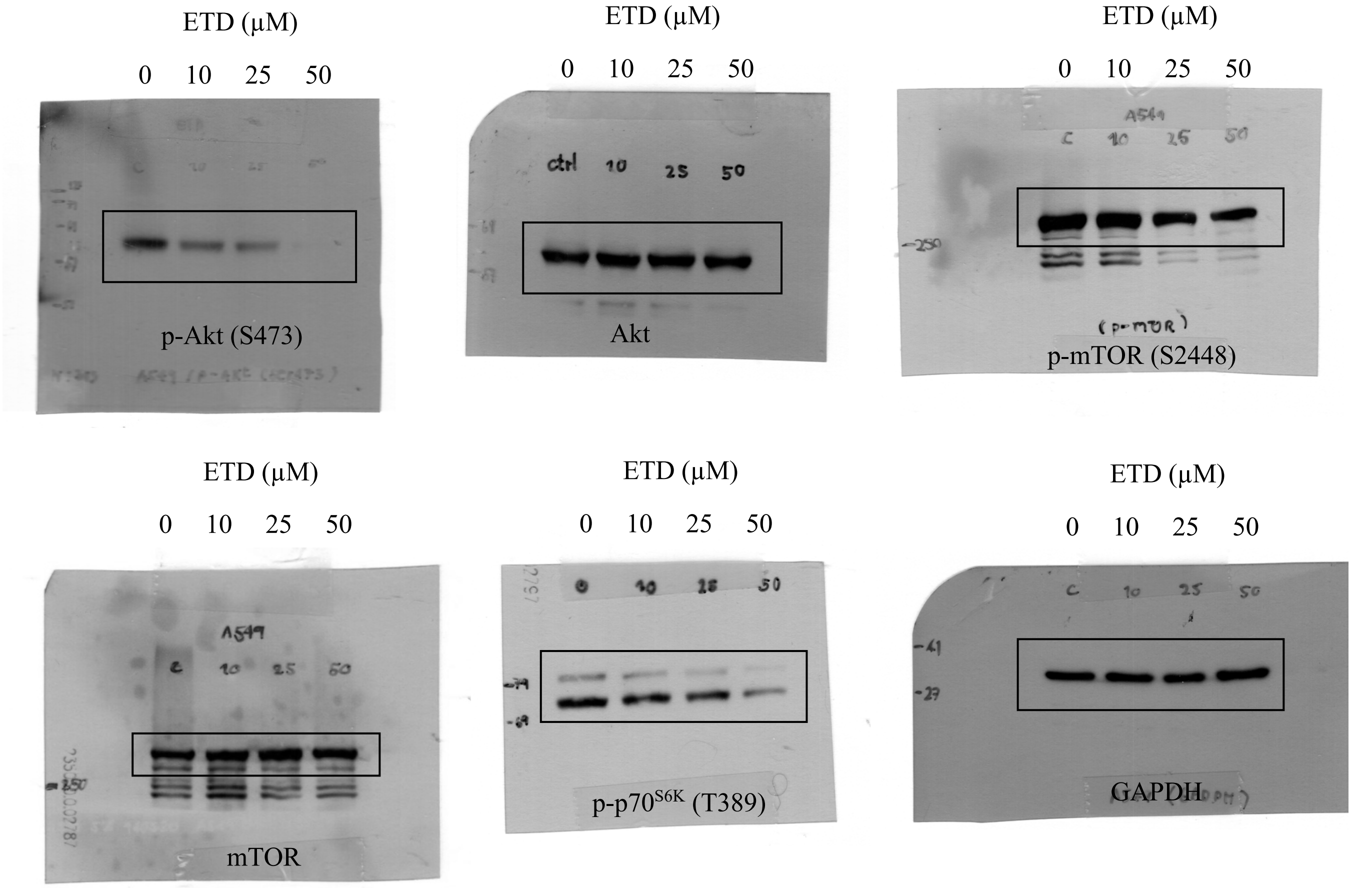

Figure S3 Uncropped western blot of A459 cells used in Fig. 4A

Supplementary Fig S4

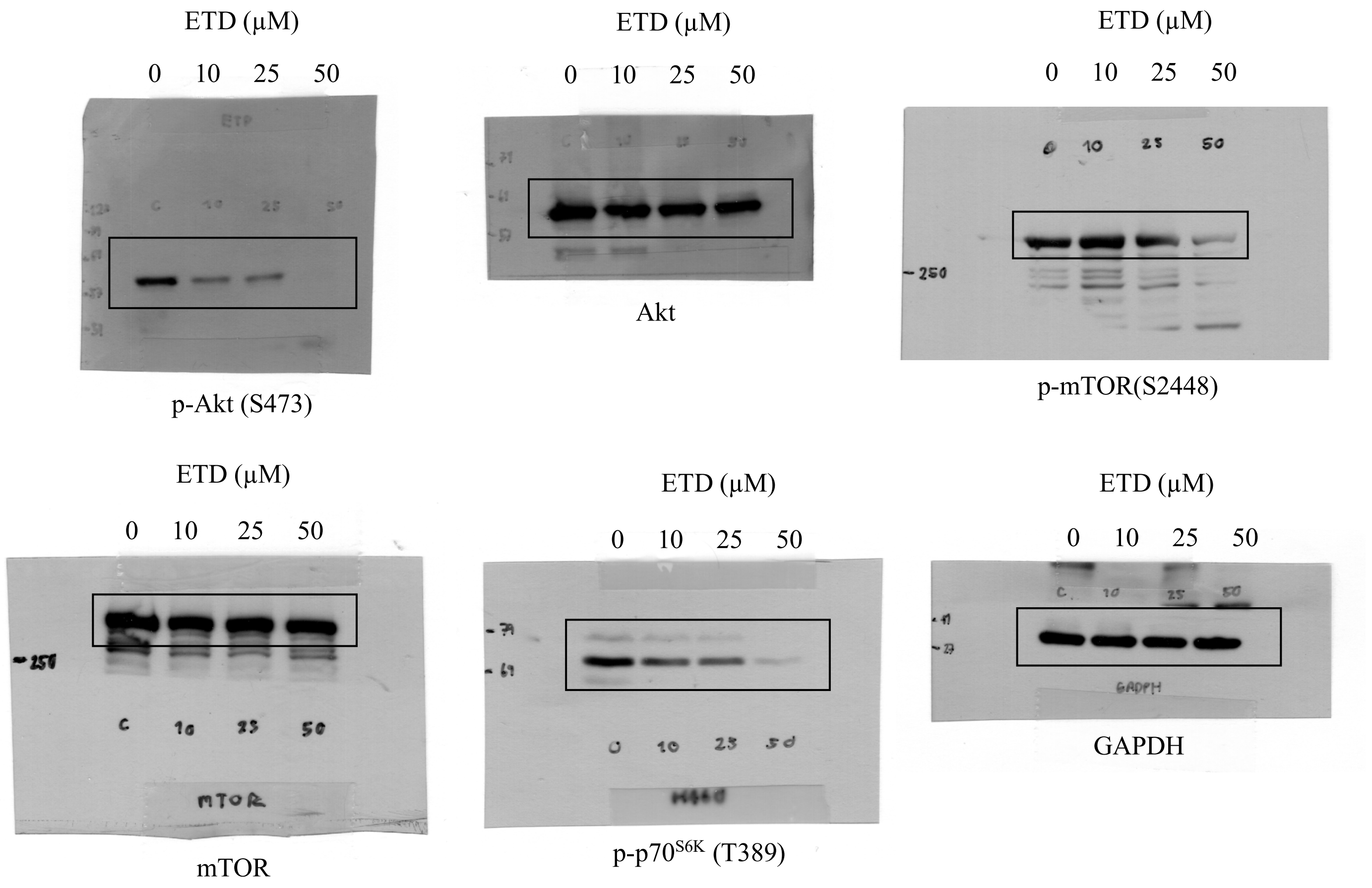

Figure S4 Uncropped western blot of H460 cells used in Fig. 4A

Supplementary Fig S5

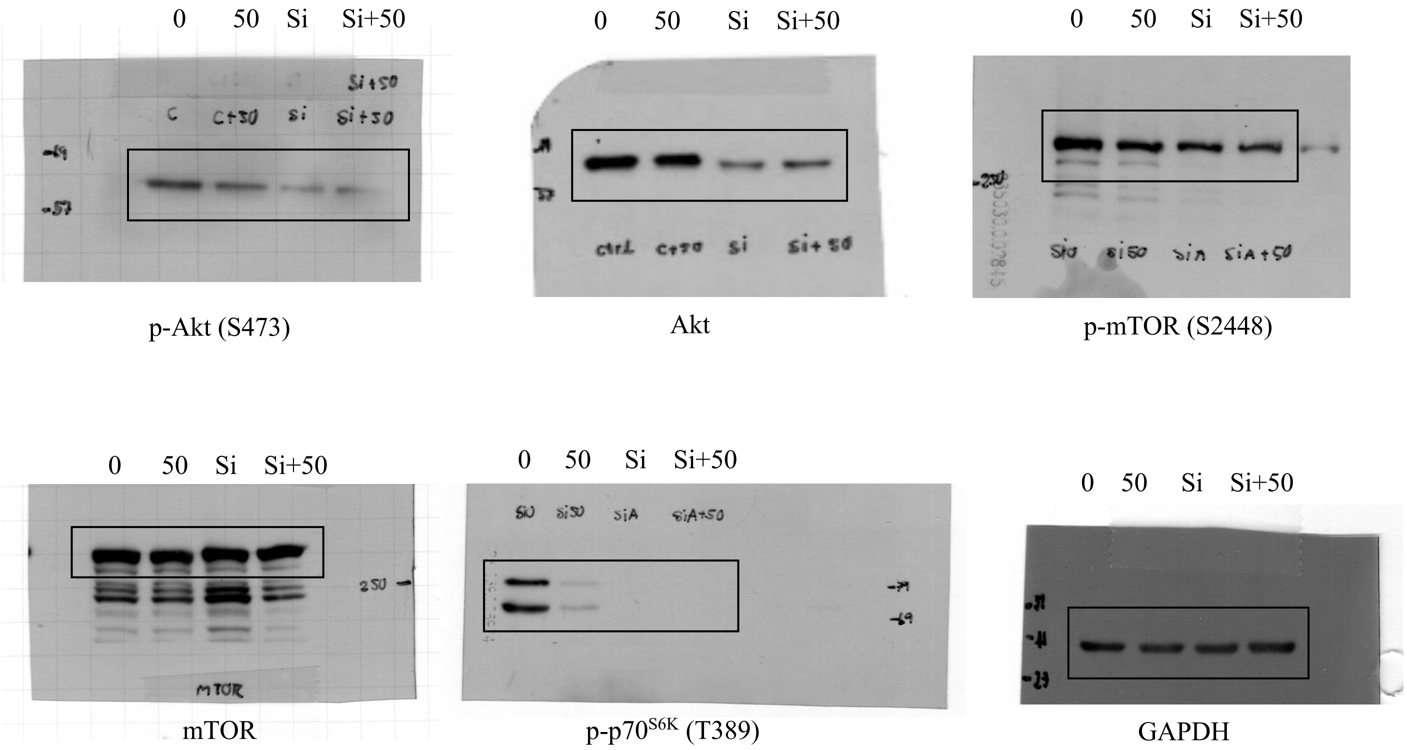

Figure S5 Uncropped western blot of A549 cells used in Fig. 4B

Supplementary Fig S6

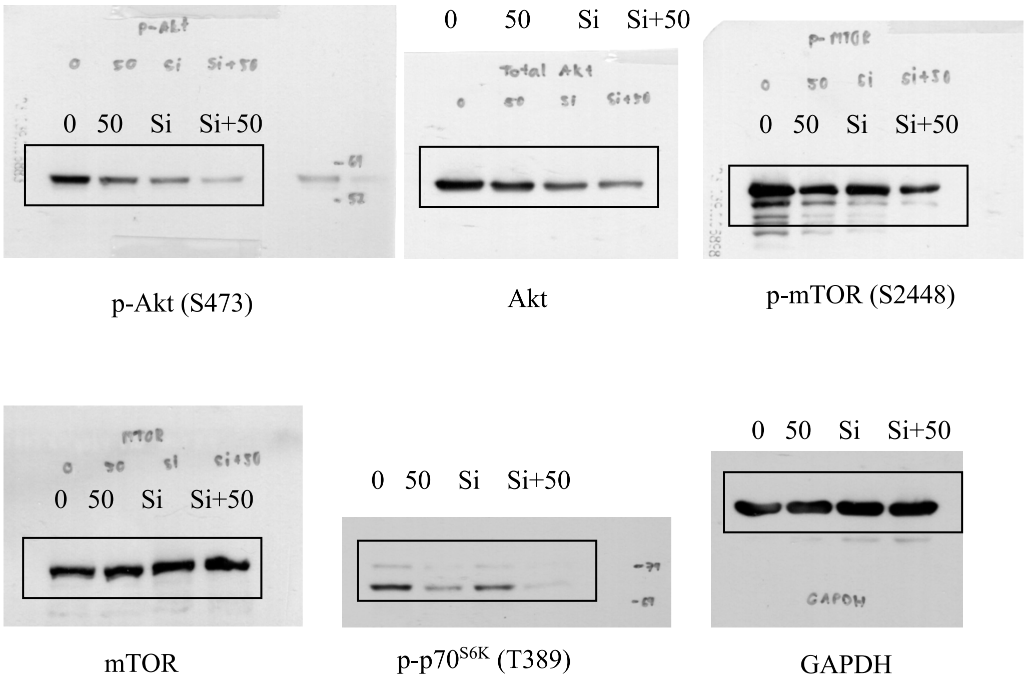

Figure S6 Uncropped western blot of H460 cells used in Fig. 4B

Supplementary Fig S7

ETD (μM)

0 50

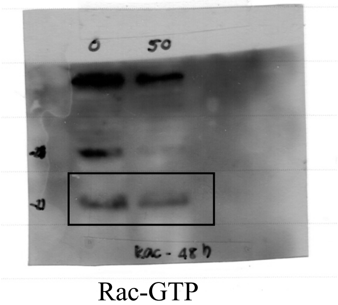

Rac-GTP

ETD (μM)

0 50

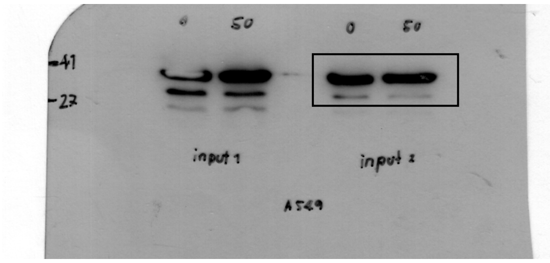

GAPDH

Figure S7 Uncropped western blot of A549 cells used in Fig. S1

Supplementary Fig S8

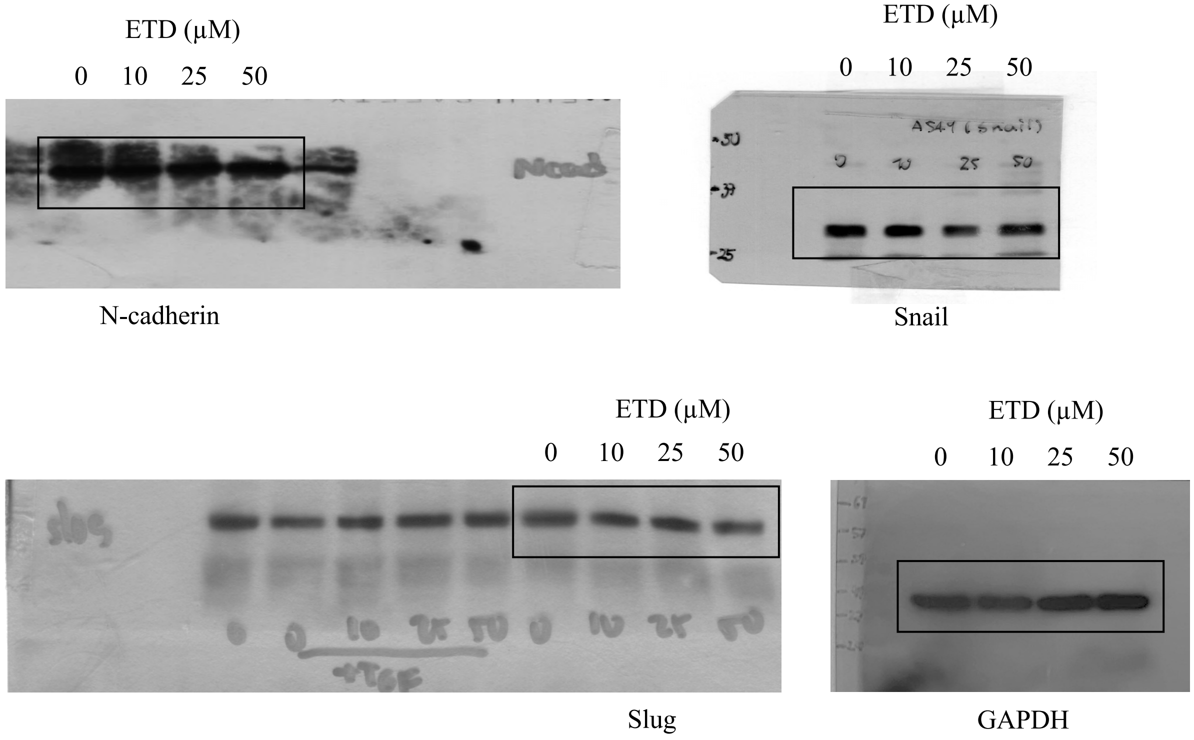

Figure S8 Uncropped western blot of A549 used in Fig. S2A

Supplementary Fig S9

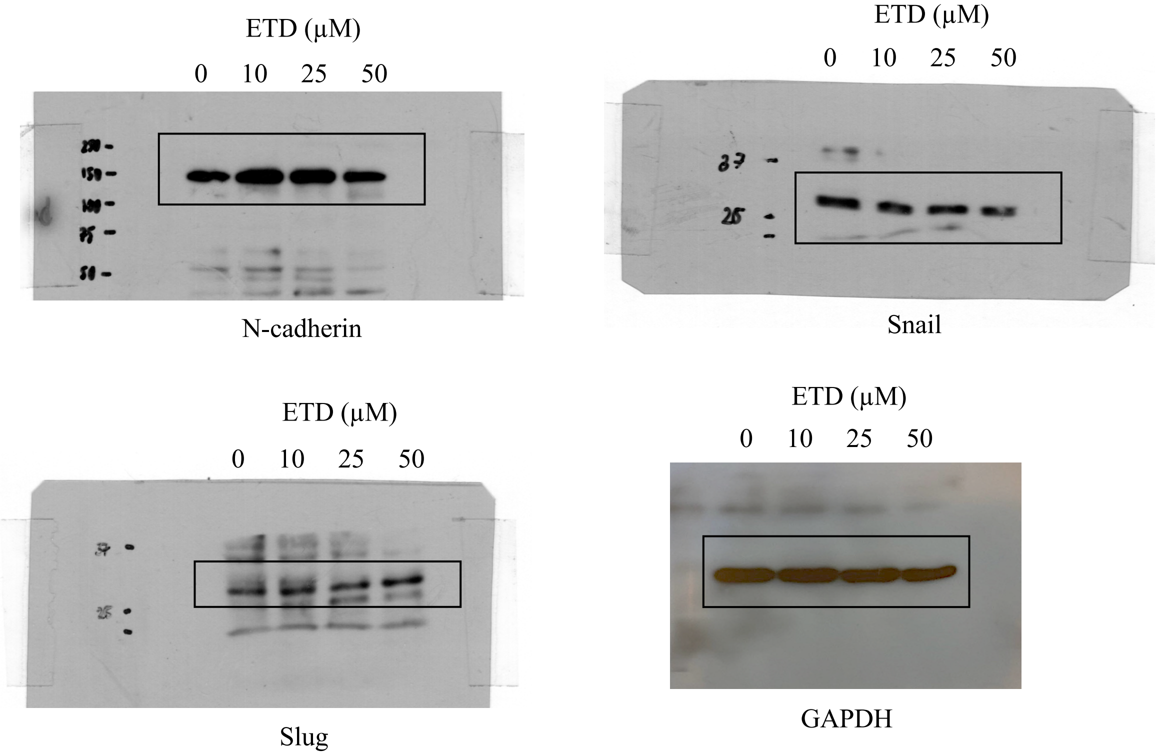

Figure S9 Uncropped western blot of H460 used in Fig. S2A

References

1. Schneider, C. A., Rasband, W. S. & Eliceiri, K. W. NIH Image to ImageJ: 25 years of image analysis. *Nat. Methods* **9**, 671–675 (2012).
